# Multi-Source Sensing of Overburden Movement, Surrounding-Rock Failure Evolution, and Mine-Pressure Response Mechanisms in a Longwall Face

**DOI:** 10.3390/s26154838

**Published:** 2026-07-31

**Authors:** Minfu Liang, Xinze Lu, Ke Hong, Huan Gong, Wei Huang, Rongwei Fan

**Affiliations:** School of Mines, China University of Mining and Technology, Xuzhou 221116, China; liangmf2014@cumt.edu.cn (M.L.); ts25020082a31@cumt.edu.cn (K.H.); ts25020131p31@cumt.edu.cn (H.G.); 01225236@cumt.edu.cn (W.H.); 06225082@cumt.edu.cn (R.F.)

**Keywords:** longwall mining, multi-source sensing, Fiber Bragg grating, overburden movement, UDEC, damage index, mine-pressure response, LSTM

## Abstract

The coupled characterization of overburden movement, surrounding-rock failure evolution, and mine-pressure response remains difficult during high-intensity longwall mining because these processes are commonly measured and interpreted using separate data streams. In this study, a multi-source sensing and interpretation framework was established for the S8310 longwall face of Yangmei No. 1 Mine by integrating physical similarity simulation, underground monitoring, UDEC numerical modeling, region-of-interest (ROI) image-feature extraction, and sliding-window long short-term memory (LSTM) analysis. Fiber Bragg grating (FBG) sensors and conventional monitoring were used to obtain key-stratum deformation and support-pressure responses. A joint-state-based damage index (DI) was constructed from fixed-ROI UDEC outputs to quantify progressive structural activation beneath the key stratum. The results indicate that the overburden evolved from global bending and local crack initiation to fracture expansion, interface degradation, and interlayer slip. In the physical model, the initial weighting interval was approximately 37.5 cm, and the periodic weighting intervals were mainly 13.3–15.7 cm; these correspond to prototype-scale distances of approximately 37.5 m and 13.3–15.7 m, respectively. Peak abutment pressure occurred approximately 9–17 cm ahead of the face at the model scale, with a peak coefficient of 1.40–1.68. When the prototype advance distance increased from 37.5 m to 80.3 m, the ROI exhibited enhanced joint activation and a transition from local slip to continuous tensile opening and slip. For the pressure-sequence analysis, the 24-step-window LSTM produced lower errors than the 36- and 48-step-window LSTM models under the same preprocessing and training settings. Additional persistence, moving-average, and random forest baselines were added to clarify the prediction context: the persistence baseline performed strongly because of the high short-term autocorrelation of the pressure series, whereas the 24-step-window LSTM outperformed the moving-average and random forest baselines. Accordingly, the LSTM module is interpreted as an auxiliary temporal-pattern identification tool rather than as an exclusive optimal predictor. The comparison between DI evolution and pressure-prediction behavior suggests that rapid DI growth is mechanically consistent with stronger pressure-sequence non-stationarity and increased prediction deviation near active mine-pressure events. The proposed framework provides a sensor-oriented and physically interpretable approach for linking overburden failure evolution with mine-pressure response in longwall mining.

## 1. Introduction

Under high-intensity longwall mining conditions, the surrounding rock around a stope undergoes pronounced structural disturbance, stress redistribution, and periodic mine-pressure manifestations [1,2,3,4]. The breakage of key strata and the associated load-transfer process govern engineering responses such as initial weighting, periodic weighting, abnormal hydraulic-support resistance, and local instability of the surrounding rock. These phenomena are direct external manifestations of overburden-structure evolution and provide essential criteria for evaluating support performance and ground-control effectiveness. Therefore, clarifying how the surrounding rock evolves from progressive deformation to localized instability, and how this structural evolution is projected into support-pressure and displacement responses, is a fundamental issue in mine-pressure theory and strata control [5,6,7,8,9].

Substantial work has been conducted on the relationship between overburden failure and mine-pressure response. Key-stratum structural models have been used to explain stress transfer and weighting events during mining [10]. Structural-instability perspectives have further clarified the link between roof failure and pressure manifestation [11]. Numerical simulations have been applied to characterize fracture propagation and stress redistribution in mined strata [12]; whereas, studies of block rotation and structural reorganization have demonstrated the controlling role of key-stratum movement in pressure response [13]. Physical similarity simulation provides controllable experimental evidence for abutment-pressure evolution and overburden movement [14], and field monitoring strengthens the correspondence between laboratory models and in situ mining conditions [15]. In recent years, optical-fiber sensing and multi-source monitoring have enabled continuous perception of key-stratum deformation and abnormal pressure evolution [16,17,18]. Data-driven models have also been introduced to predict mine-pressure trends and improve the timeliness of abnormal-event recognition [19].

Despite these advances, several limitations remain in the unified characterization of surrounding-rock structural evolution and mine-pressure response. Existing studies often analyze fracture development, support-pressure fluctuation, or displacement response separately, and a unified framework capable of linking fracture expansion, interface degradation, interlayer slip, and mine-pressure anomalies remains insufficient [20,21,22]. In addition, numerical simulation, physical similarity testing, and field monitoring are frequently used as independent tools, with limited cross-scale comparison under the same coordinate system and indicator definition. Consequently, the relationship between structural evolution and mine-pressure manifestation is often interpreted qualitatively. Although some data-driven studies can fit or forecast pressure sequences, they commonly rely directly on historical time-series information and lack physical constraints associated with key-stratum breakage and structural reorganization. The mechanism chain from surrounding rock failure evolution to abnormal mine-pressure manifestation requires further quantification and validation [23,24,25].

**Figure 1:**
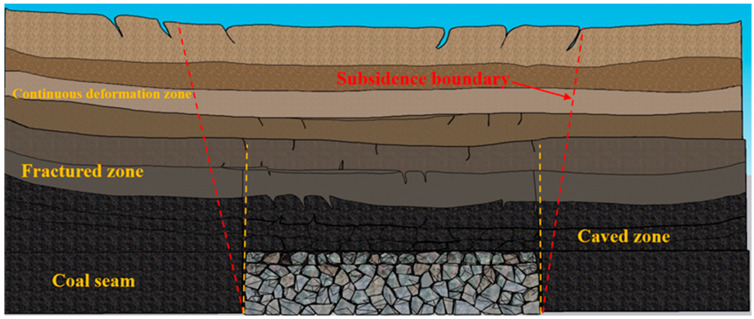
Schematic diagram of overburden-structure evolution and mine-pressure response. The red dashed lines denote the subsidence boundaries, and the colored labels identify the caved zone, fractured zone, and continuous-deformation zone.

To address these issues, this study takes the S8310 longwall face of Yangmei No. 1 Mine as the engineering background and constructs a multi-source sensing framework around the mechanism chain of key-stratum breakage, load redistribution, and mine-pressure response. First, physical similarity simulation and FBG sensing are used to obtain multi-source responses of key-stratum deformation and support pressure, and underground monitoring data are used to identify staged mine-pressure characteristics. Second, joint-state transition features were extracted from UDEC Joint Plane State outputs within a fixed ROI beneath the key stratum, and a joint-state-based damage index was defined to quantify progressive structural activation along the advance direction. Shear-displacement and stress-response maps were further used as mechanical corroboration for the same damage-evolution process. Finally, a sliding-window LSTM model is introduced to predict the support-pressure sequence and test whether the mine-pressure response contains learnable patterns constrained by structural evolution. The overall mechanism chain and multi-source sensing framework are illustrated in Figure 1.

## 2. Materials and Methods

### 2.1. FBG Sensing and the Multi-Source Data System

To clarify the sensing chain, an FBG sensor does not directly measure displacement or pressure; instead, it measures the shift in the reflected Bragg wavelength [26]. The Bragg condition is expressed as

(1) $$\lambda_{B}={2n}_{\mathrm{eff}}\Lambda ,$$

where λ_B_ is the reflected Bragg wavelength, n_eff_ is the effective refractive index of the fiber core, and Λ is the grating period. Under coupled strain–temperature action, the wavelength shift can be expressed as

(2) $$\frac{{\Delta \lambda }_{B}}{\lambda_{B}}{=(1-p}_{e})\varepsilon +(\alpha +\xi )\Delta T,$$

where p_e_ is the effective photo-elastic coefficient, ε is the axial strain, α is the thermal expansion coefficient, ξ is the thermo-optic coefficient, and ΔT is the temperature variation [27,28,29].

In the physical model test, FBG1–FBG5 were arranged in the coal-seam floor to characterize the evolution of floor abutment pressure, whereas FBG6–FBG7 were vertically embedded near the first sub-key stratum to capture key-stratum deformation and breakage precursors. The packaged FBG sensors were developed and fabricated in-house at China University of Mining and Technology (Xuzhou, China). Therefore, the measured wavelength drift was first converted into strain through the calibrated FBG strain model and then interpreted as a pressure or deformation response according to the sensor packaging structure and calibration relationship. The key-stratum response reported in this study should therefore be understood as an FBG-derived deformation/strain response rather than a direct optical measurement of displacement.

**Figure 2:**
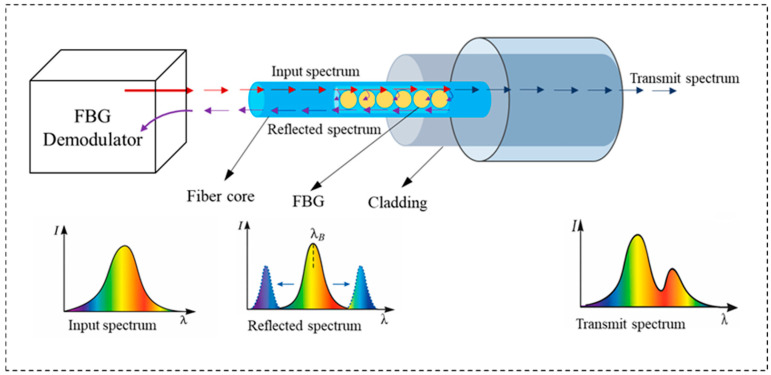
Sensing principle of a Fiber Bragg grating sensor. The arrows indicate the incident, reflected, and transmitted optical paths, and the colored curves represent the corresponding optical spectra.

Temperature compensation was carried out using a strain-free reference grating placed in the same thermal environment as the loaded grating. The sensing principle of the FBG sensor is illustrated in Figure 2. For the self-developed hydraulic-support pressure sensor fabricated at China University of Mining and Technology (Xuzhou, China) used as the pressure-conversion element, the absolute values of the temperature-compensated pressure sensitivities of the two pressure-sensor gratings were 0.0351 and 0.0346 nm/MPa, respectively, which were very close to the absolute constant-temperature sensitivity values of 0.0352 and 0.0348 nm/MPa. The residual sensitivity differences after compensation were approximately 0.28% and 0.57%, indicating that the reference-grating compensation effectively removed most of the temperature-induced drift. The calibration and temperature-compensation results are summarized in Table 1.

The hydraulic-support pressure sensor was mechanically calibrated through repeated loading and unloading. Before calibration, the sensor was preloaded and unloaded until the wavelength response became stable. At 20 °C, the initial wavelengths of the measuring and temperature-compensation gratings were 1529.621/1532.286 nm for grating group 1 and 1534.926/1537.545 nm for grating group 2, respectively. Pressure was then applied from 0 to 25 MPa in increments of 5 MPa for five loading–unloading cycles. The coefficients of determination for the loading/unloading processes were 0.9999/0.9998 for grating group 1 and 0.9997/0.9996 for grating group 2, confirming that the pressure-to-wavelength conversion remained approximately linear within the calibration range.

The physical similarity experiment used an FBG intelligent sensing method to conduct synchronous multi-point monitoring [30,31,32]. Multiple gratings with distinct reflected center wavelengths were written along the same fiber to form a quasi-distributed sensing array. To ensure stable demodulation, the center wavelengths and their expected drift ranges were designed to avoid spectral overlap between adjacent gratings. This spacing constraint was incorporated into the sensing-system design to determine the number of sensing points, center-wavelength allocation, and maximum allowable wavelength drift, thereby serving as a prerequisite for data-quality control [33,34].

### 2.2. ROI-Based UDEC Image-Feature Extraction and Interpretable Damage Index

To provide a reproducible damage representation from the UDEC 7.0 (Itasca Consulting Group, Inc., Minneapolis, MN, USA) results, the damage index (DI) was defined as an ROI-based image-derived descriptor from the exported joint-plane-state maps. The DI does not replace the native UDEC mechanical variables; instead, it provides a unified regional measure of joint-state activation within the same physical window. The fixed ROI was located in the main damage channel above the gob and below the key stratum, so that all advance stages were evaluated using an identical spatial criterion.

In this study, the quantitative DI was calculated from the Joint Plane State maps and is therefore referred to as a joint-state-based DI. The shear-displacement and stress-response maps were used as mechanical corroboration for the same damage-evolution process rather than as additional numerical components of this specific DI curve. This treatment ensures that the DI calculation remains consistent with the available UDEC outputs and avoids mixing variables with different physical dimensions.

Only colored joint-state pixels within the fixed ROI were used for DI calculation. Black and gray line features were excluded because the exported UDEC maps contain both joint-state marks and block or mesh boundaries, which may otherwise lead to over-counting of inactive structural features. The resulting state-based damage score Q(s) was calculated as follows:

(3) $$Q(s)=0.45N_{slip-p}+0.70N_{tens-slip}+1.00N_{slip-tens}+0.85N_{tens}$$

where *N* denotes the number of classified pixels for each joint-state category within the fixed ROI. The normalized damage index was then calculated as follows:

(4) $$\mathrm{DI}(s)=\frac{Q(s)}{\max[Q(s)]}$$

where max[Q(s)] is the maximum state-based damage score among the selected advance stages. Therefore, DI(s) = 1.00 represents the maximum damage level within the selected advance sequence rather than an absolute physical failure ratio of 100%. The pixel-classification and weighting rules used for the joint-state-based DI are summarized in Table 2.

The resulting DI values and the corresponding physical-model events are listed in Table 3.

The sensitivity-analysis results under alternative weighting schemes are presented in Table 4.

**Figure 3:**
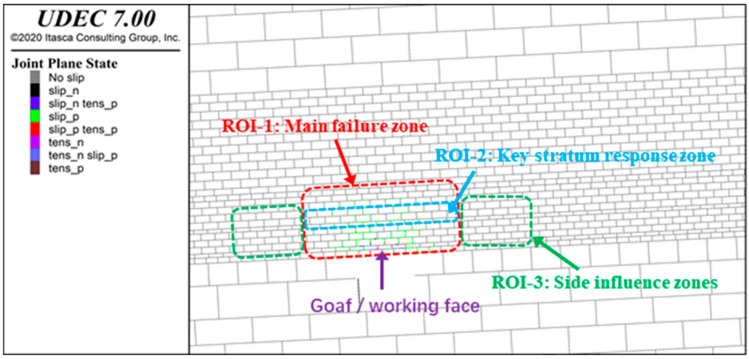
Example ROI definition for UDEC image-feature extraction at an advance distance of 37.5 m. ROI-1, enclosed by the red dashed box, denotes the main failure zone used for DI calculation; ROI-2 denotes the key-stratum response zone; ROI-3 denotes the side influence zones; and the purple arrow indicates the gob/working-face region.

The sensitivity results show that changes in the weighting strategy altered the absolute DI values at the early stages, but did not change the main evolutionary conclusion. Under all tested weighting schemes, DI remained relatively low at 37.5–51.3 m, increased sharply by 67.0 m, and reached the maximum at 80.3 m. Therefore, the identified damage-activation interval is controlled by the joint-state evolution within the ROI rather than by a single arbitrary weight setting. The ROI definition at an advance distance of 37.5 m is shown in Figure 3.

**Figure 4:**
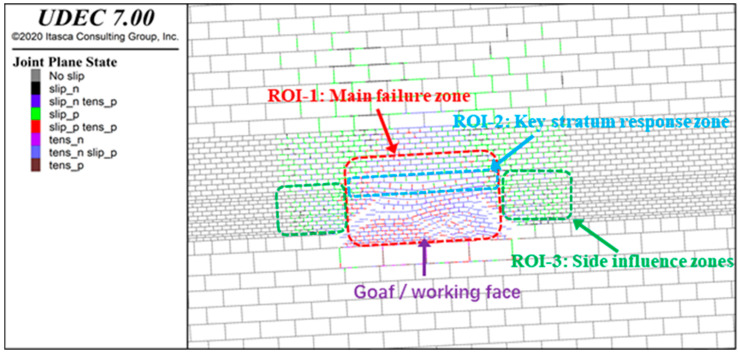
Example ROI definition for UDEC image-feature extraction at an advance distance of 80.3 m. ROI-1, enclosed by the red dashed box, denotes the main failure zone used for DI calculation; ROI-2 denotes the key-stratum response zone; ROI-3 denotes the side influence zones; and the purple arrow indicates the gob/working-face region.

For comparison, the ROI definition at an advance distance of 80.3 m is shown in Figure 4.

### 2.3. Physical Similarity Simulation and FBG Monitoring Arrangement

The physical similarity model was established for approximately 115 m of prototype strata. With a geometric similarity ratio of 1:100, the corresponding model height was 115 cm, and the model frame dimensions were 250 cm × 20 cm × 115 cm. The advance distance s was used as the unified comparison axis. Therefore, the characteristic positions observed in the physical simulation at 37.5, 51.3, 67.0, and 80.3 cm were converted to the corresponding prototype-scale positions of 37.5, 51.3, 67.0, and 80.3 m, respectively. The similarity rules adopted in the physical model are summarized in Table 5.

The mix codes in Table 6 are material-ratio codes rather than independent lithological symbols. The physical model was prepared using sand as the aggregate, industrial gypsum and industrial calcium carbonate as cementing materials, water as the mixing medium, and mica powder to simulate bedding interfaces. Therefore, the mix codes record the relative proportion levels of the similar-material components used for different lithologies. The material-ratio codes and the functions of the constituent materials are explained in Table 6.

**Figure 5:**
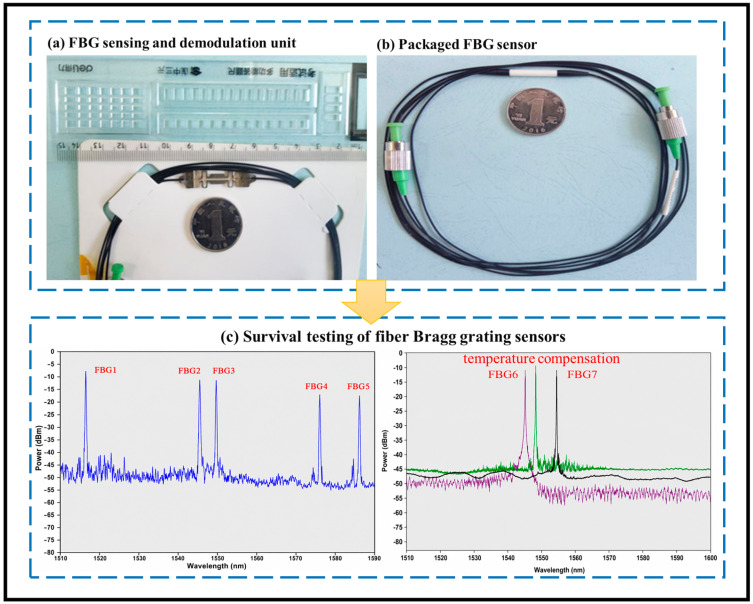
FBG sensing system and survival-test results: (**a**) FBG sensing and demodulation unit; (**b**) packaged FBG sensor; and (**c**) survival-test spectra of FBG1–FBG7. The arrows indicate the sensing and testing sequence, and the colored curves distinguish the sensing and temperature-compensation channels.

A physical similarity model was constructed according to the geological conditions of the S8310 large-mining-height longwall face at Yangmei No. 1 Mine. Seven FBG sensors were embedded in the model, and conventional monitoring devices were used to obtain multi-source observations of surrounding-rock structural evolution. The model was designed around the main process of key-stratum breakage, load redistribution, and support response. The FBG sensing equipment and the sensor survival-test results are shown in Figure 5.

The detailed lithological sequence, material-ratio codes, layer thicknesses, and material masses used in the physical similarity model are summarized in Table 7.

**Figure 6:**
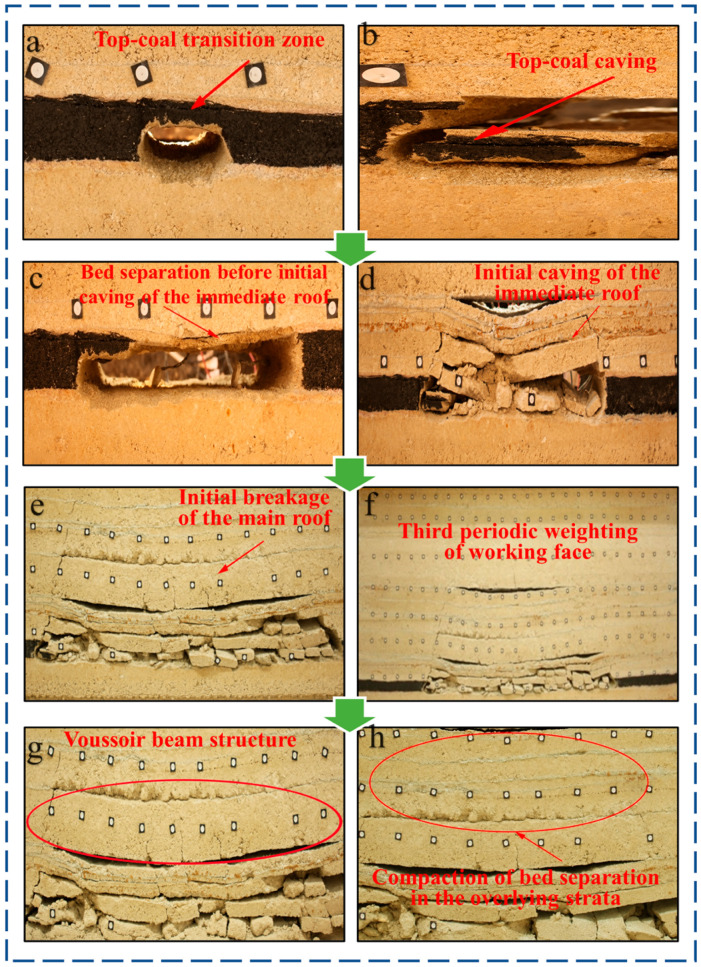
Progressive failure process of the roof structure in the physical similarity model: (**a**) top-coal transition zone; (**b**) top-coal caving; (**c**) bed separation before the initial caving of the immediate roof; (**d**) initial caving of the immediate roof; (**e**) initial breakage of the main roof; (**f**) third periodic weighting of the working face; (**g**) formation of the voussoir-beam structure; and (**h**) compaction of bed separation in the overlying strata. The green arrows indicate the temporal evolution sequence, whereas the red arrows identify the characteristic roof-failure features.

The physical similarity simulation followed the working-face advance process and focused on the sequence of “key-stratum breakage-load redistribution-support response”. Under the premise of satisfying geometric similarity, load similarity, and material-mechanical similarity, the similarity ratio was determined and controllable similar materials were selected so that the layered structure, mining disturbance, and macroscopic breakage morphology in the model statistically corresponded to the underground prototype. To reduce the influence of boundary effects on the breakage morphology, the model boundaries were reinforced and buffered. The advance distance s was used as the only spatial physical variable to ensure that the data from different sensing systems corresponded on the same “space-time axis”. The progressive failure process of the roof structure is shown in Figure 6.

## 3. Analysis of Experimental Results

### 3.1. Analysis of Key Results from the Physical Similarity Simulation

(1) Overburden Breakage and Structural Evolution

Top-coal caving in the transition zone: during the transition from the open-off cut to normal mining height, the top coal underwent overall instability and caving when the working face advanced to approximately 13.6 cm from the open-off cut. The caving length in the physical model was approximately 14.4 cm, and the caving angle was approximately 70°.

Bed separation and initial caving of the immediate roof: when the face advanced to approximately 15.5 cm, a bed-separation fracture appeared between the immediate roof and the main roof, with a length of approximately 10.8 cm that increased with advance. When the face advanced to approximately 19 cm, the immediate roof experienced its first local caving. Subsequently, the exposed immediate roof underwent overall caving, with a caving length of approximately 17.5 cm. The immediate-roof caving process showed an evolution from layered caving to continuous caving, and the caving angle was generally approximately 60–75°.

Initial and periodic weighting of the main roof: the initial breakage occurred when the face advanced to approximately 37.5 cm from the open-off cut, corresponding to an initial weighting interval of approximately 37.5 cm and a breakage angle of approximately 70°. Stable periodic breakage then occurred at advance distances of 51.3, 67.0, 80.3, and 95.0 cm, with periodic weighting intervals concentrated between 13.3 and 15.7 cm and an average of approximately 14.3 cm.

During the enlargement of bed separation and periodic breakage, the main roof gradually formed and evolved into a “voussoir beam” structure. Periodic weighting corresponded to repeated key-block rotational instability and progressive compaction of the gob. The caving characteristics of the immediate roof are summarized in Table 8.

(2) Distribution of Advance Abutment Pressure

As the working face advanced and as the key stratum underwent initial and periodic breakage, the caved rock mass in the gob was gradually compacted and stabilized, and the peak of the advance abutment pressure migrated forward as a whole.

Peak position and intensity: the peak abutment pressure was located approximately 9–17 cm ahead of the working face, and the peak intensity was approximately 1.40–1.68 times the original rock stress.

Influence range: the advance influence range was approximately 36–90 cm.

(3) Deformation and Movement of Key Strata

First sub-key stratum: local subsidence occurred when the face advanced to approximately 28 cm, and overall caving occurred at 37.5 cm, corresponding to initial weighting. The maximum subsidence was approximately 47 mm. After entering the periodic weighting stage, periodic breakage occurred, and the maximum deformation generally decreased with further advance.

**Figure 7:**
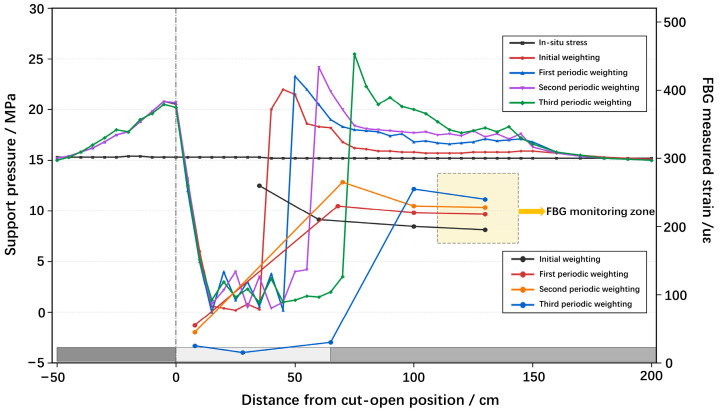
Distribution of abutment pressure during working-face excavation. The black horizontal line denotes the in situ stress; the colored curves denote the initial and periodic weighting responses; and the yellow shaded region indicates the FBG monitoring zone.

Second sub-key stratum: subsidence began and propagated forward when the face advanced to approximately 80.3 cm. The deformation then gradually increased and stabilized at approximately 43 mm. The combined abutment-pressure and FBG-derived strain responses are shown in Figure 7.

### 3.2. FBG Monitoring System and Data Processing

FBG monitoring used wavelength drift as the strain response, and temperature compensation was applied to suppress the interference of environmental temperature variation on the measurements. In this experiment, a multi-point series FBG array was used to monitor floor abutment pressure and key-stratum deformation response synchronously. Specifically, FBG1-FBG5 were used to characterize the spatial distribution of floor abutment pressure and its evolution with face advance; whereas, FBG6-FBG7 were arranged near the key strata to capture key-stratum deformation and precursors of breakage. The raw data were resampled and aggregated according to the advance distance s. Within the window corresponding to each advance step, the mean, peak, percentile, and other statistics were calculated and annotated on the same coordinate axis together with event points in the support-pressure curve, such as initial weighting and periodic-weighting peaks, to ensure comparability with the subsequent numerical simulation.

**Figure 8:**
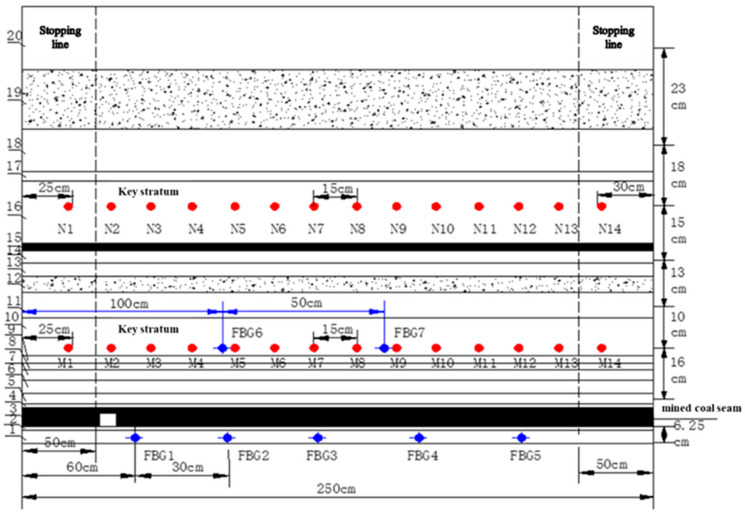
Layout of the model test system. Red points N1–N14 denote the conventional monitoring points, blue points FBG1–FBG7 denote the FBG sensing locations, the dashed vertical lines indicate the stoping boundaries, and the black layer denotes the mined coal seam.

The results show that the model exhibited a typical response sequence of “initial weighting-periodic weighting” during advance. The initial weighting interval was approximately 37.5 cm, and the subsequent periodic weighting intervals were concentrated within 13.3–15.7 cm, indicating that the key stratum evolved from overall bearing to segmented breakage under mining disturbance. The layout of the model test system is shown in Figure 8.

### 3.3. Comparison of Multi-Point Response Characteristics of Working-Face Abutment Pressure

To verify whether the rules of “key-stratum breakage, structural control, and abnormal advance bearing” identified in the physical similarity test are supported by field measurements, underground working-face monitoring data were analyzed for comparison. Unlike the physical similarity test, which focused on reproducing the breakage process under controlled conditions, field monitoring was affected by uncontrollable factors such as geological heterogeneity, production disturbance, and observational noise; therefore, it was defined as real external data. To ensure comparability between simulation and field measurements, the field data were not analyzed using acquisition time as the main axis. Instead, the pressure sequence was mapped to advance distance s, and statistics such as the mean, peak, and percentile were extracted using the same window-statistics method as in the physical similarity test. Initial and periodic weighting event points were also annotated on the same s axis. This produced a comparison framework with the same coordinate axis, the same indicator framework, and different observation conditions, allowing the mechanistic interpretation from the physical similarity test to be examined using field data.

**Figure 9:**
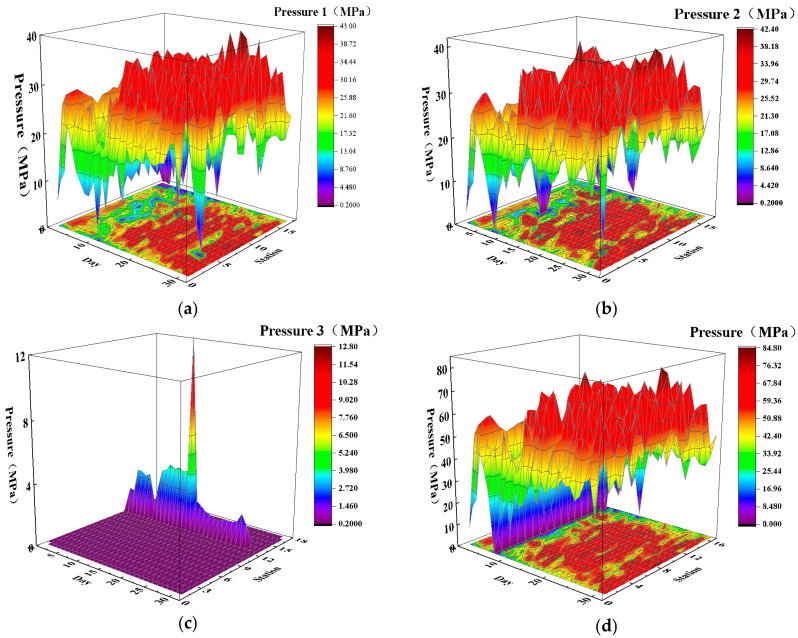
Distribution of abutment pressure. (**a**) Three-dimensional spatiotemporal distribution of support pressure at P1; (**b**) three-dimensional spatiotemporal distribution of support pressure at P2; (**c**) three-dimensional spatiotemporal distribution of support pressure at P3; (**d**) three-dimensional spatiotemporal distribution of total support pressure.

Three abutment-pressure measuring points, P1–P3, were arranged within the influence range of the working face. The pressure sequences were aligned by advance step and statistically extracted using the advance distance s as the unified coordinate. The results show that, as the working face approached, all measuring points exhibited a typical evolution pattern in which pressure gradually increased, reached a peak near the approach stage, and then decreased rapidly. This reflected the full process of formation, intensification, and attenuation of the advance bearing zone. The pressure peaks were concentrated approximately 15.4 cm ahead of the working face, and the peak intensity coefficient was approximately 1.49, indicating a distinct stress concentration zone ahead of the face whose position migrated as a whole with advance. Recognizable phase differences in the peak-occurrence steps among different measuring points provided an experimental basis for correlating mine-pressure peaks with key-stratum breakage stages. From the perspective of structural mechanics, this rule can be interpreted as the external response of the key stratum evolving from overall bending to fracture coalescence and block rotation. Initial weighting corresponds to the scale effect caused by the first through-going fracture, whereas periodic weighting corresponds to repeated instability and reorganization of the block structure. Meanwhile, FBG-derived deformation monitoring near the key strata showed alternating slow growth and staged acceleration. Before initial weighting, the deformation-response slope and fluctuation amplitude increased simultaneously, indicating parallel development of fracture propagation and stiffness degradation. When initial weighting occurred, the deformation response exhibited an abrupt jump and was aligned with multi-point pressure peaks along the advance step. The three-dimensional spatiotemporal pressure distributions at P1–P3 and the total support pressure are shown in Figure 9.

A comparison among the three points shows that the rising section of the P1 curve was shorter and the peak was sharper, indicating that near-field bearing was more sensitive to working-face advance and roof-structure adjustment. Although the peak amplitudes of P2 and P3 were relatively convergent, their peak occurrence was delayed in terms of advance step, reflecting the propagation effect of the bearing concentration zone as it migrated forward.

## 4. Characterization of Overburden Damage Evolution Based on UDEC and Its Mine-Pressure Response Mechanism

**Figure 10:**
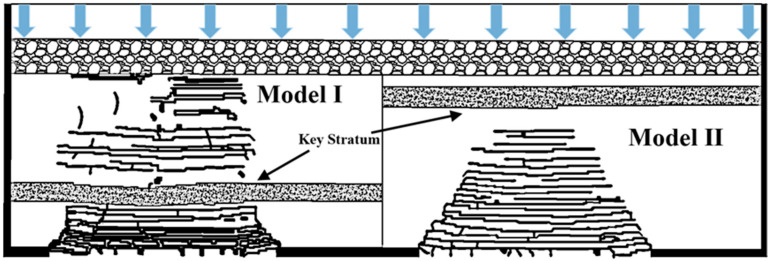
Evolution of overburden movement. Model I and Model II represent the structural states before and after key-stratum breakage and block reorganization, respectively.

The overburden above a working face is generally a layered system composed of the immediate roof, main roof, and higher strata, and its movement law is closely related to the key strata [35,36]. A key stratum is a high-stiffness stratum group that dominates overall movement and load transfer at a specific scale. As the mining span increases, the overburden response gradually changes from overall bending to coordinated deformation among layers, while bending moment and shear force accumulate rapidly within the key stratum. When the critical condition jointly determined by span, equivalent stiffness of the key stratum, and overburden load is reached, the key stratum first undergoes tensile cracking and bending failure, forming a block structure characterized by articulation, as shown in Figure 10. Under this controlling effect, the overlying secondary strata undergo secondary bed separation and fracture coalescence. The load path transfers from the immediate roof to the two ribs and the supports, and manifests as the spatiotemporal alternation of initial weighting and periodic weighting. From a structural perspective, initial weighting can be defined as the condition in which the bending-dominated failure criterion of the cantilevered main roof or basic roof is satisfied during mining advance; that is, the maximum tensile stress caused by bending in the cantilever or composite beam reaches the tensile strength of the rock mass, or the deflection reaches the instability threshold, leading to the first through-going fracture and an obvious bearing-displacement mutation. This criticality can be explained using key-stratum theory: when the controlling key stratum breaks and forms hinged blocks in the gob, the system enters a stress state characterized by a voussoir beam or three-hinged arch [37,38]. Periodic weighting corresponds to repeated breakage and block reorganization of the key stratum and the underlying layered strata. It appears as either regular periodicity or alternating strong and weak weighting, with the intensity and interval jointly controlled by roof thickness, mechanical parameters, joint development, and compaction degree of the caving zone. When working-face advance causes the exposed span of the main roof to reach the critical condition, the main roof, namely the first key stratum, undergoes the first through-going failure and large-scale collapse, usually accompanied by considerable roof subsidence and significant dynamic loading. Before collapse, the main roof gradually accumulates bending deformation under the constraints of the supports, coal pillars at both ends, and the coal wall, behaving as a cantilever beam or near-arch structure. Once the material or stability limit is reached, brittle failure occurs. The initial caving interval is generally significantly larger than subsequent periodic weighting intervals [39,40].

### 4.1. UDEC Fracture Evolution and Advance-Stage Analysis

To improve reproducibility, the UDEC calculation is described using the same advance-distance coordinate as the physical model. The numerical model was solved using UDEC 7.00. Coal-seam excavation started from the open-off cut and proceeded in equal excavation steps. According to the excavation record, the working face was advanced in 10 m increments, and the saved states closest to the physical-model characteristic positions were used to represent s = 37.5, 51.3, 67.0, and 80.3 m. Therefore, the mechanical interpretation focuses on stage correspondence rather than exact point-to-point equality between every numerical excavation step and every physical-model observation. The main UDEC model settings used for reproducibility are summarized in Table 9.

The cross-scale correspondence between the physical-simulation and UDEC stages is presented in Table 10.

This cross-scale comparison was used as the verification basis for the numerical interpretation. The UDEC model was not evaluated by matching a single image only; instead, the selected stages were required to reproduce the physical sequence of initial weighting, periodic weighting, damage expansion, and key-stratum response. The agreement in event order and damage-location evolution supports the use of the UDEC results for mechanism interpretation.

Based on the dual observation framework of “controllable simulation-field external validation” constructed from the physical similarity test and underground measurements, stable features of overburden breakage and mine-pressure response along the advance-distance axis were identified. These features included the stage division of initial and periodic weighting, formation of the advance abutment-pressure peak, and key-stratum displacement responses before and after breakage. Working-face mine-pressure anomalies were not merely pressure fluctuations, but were associated with key-stratum fracture propagation and structural coalescence. On the basis of the preceding physical similarity simulation and field monitoring, UDEC numerical simulation was used to analyze fracture propagation, shear slip, stiffness degradation, and structural state to reveal the relationship between overburden-structure breakage and abutment-pressure anomalies during mining. Based on the ROI extraction and DI construction methods described in Section 2, the key stratum and its adjacent dominant failure-channel area were defined as a fixed analysis window. Pixel statistics and representative-variable extraction were performed on UDEC images under different advance steps, forming a continuous damage-evolution sequence along the advance-distance axis. The UDEC images indicate that this region simultaneously exhibited concentrated fracture expansion, obvious contact degradation, and activated interfacial dislocation, giving it clear mechanical relevance. Four typical advance stages, 37.5, 51.3, 67.0, and 80.3 m, were selected for comparative analysis. The former represents a stage in which local damage had formed beneath the key stratum but had not fully expanded, with the surrounding-rock response dominated by deformation accumulation and local crack initiation. The latter stages correspond to further damage concentration, interface degradation, and enhanced dislocation, which are suitable for revealing the key transition of the surrounding rock from progressive deterioration to significant activation. Compared with a single advance step, comparison between these stages more clearly identifies the evolutionary path from “local disturbance” to “substantial weakening” of the structural state.

### 4.2. Damage Activation in the ROI and Its Correspondence with Mine-Pressure Response

On the basis of the initial weighting, periodic weighting, and support-pressure peak migration laws identified by physical similarity simulation and field monitoring, this section uses the UDEC joint-state results to analyze the spatial evolution of surrounding-rock damage beneath the key stratum. The preceding results show that initial breakage of the main roof occurred near a model advance distance of 37.5 cm, followed by periodic breakage at 51.3, 67.0, and 80.3 cm. Because the geometric similarity ratio of the physical simulation was 1:100, these characteristic positions were converted to prototype scale and corresponded to advance distances of s = 37.5, 51.3, 67.0, and 80.3 m. Accordingly, the four corresponding typical stages in the UDEC model were selected for joint-state comparison. Figure 9 shows the evolution characteristics of overburden joint states at different advance distances. The red dashed box in the figure represents the fixed ROI, which was located in the main damage zone from the immediate roof above the gob to the lower part of the main roof. This region covered the spatial range with the most concentrated joint slip, tensile failure, and combined slip–tensile failure, and served as the unified statistical window for subsequent DI calculation and correlation analysis with mine-pressure response. The purpose of using a fixed ROI was to ensure a consistent damage-statistics criterion across different advance stages and to avoid incomparability caused by changes in the observation range.

When the working face advanced to s = 37.5 m, obvious joint-state activation had already appeared within the ROI above the gob, although failure remained mainly confined to the immediate roof and the gob boundary. Green slip states and a small number of tensile-related states began to appear, indicating that the roof structure during initial weighting had changed from overall bending to local breakage and interface activation. This phenomenon corresponded to the physical similarity simulation result in which initial main-roof breakage occurred near 37.5 cm, indicating that initial mine-pressure manifestation was not caused solely by overall roof subsidence but occurred synchronously with joint slip and local tensile failure in the immediate roof and the lower part of the main roof.

When the advance distance increased to s = 51.3 m, the range of joint activation within the ROI further expanded, and the slip state showed a more continuous distribution above the gob. Compared with the s = 37.5 m stage, the failure zone had extended from the gob boundary into the overlying strata, and the overburden structure after initial breakage began further adjustment. This stage corresponded to the first periodic breakage position in the physical similarity simulation, indicating that the formation of periodic weighting was closely related to the expansion of the joint-slip range above the gob and the reduction in the bearing capacity of structural planes.

After the face advanced to s = 67.0 m, the joint state within the ROI changed from local activation to large-scale continuous activation. Slip, tensile failure, and combined slip–tensile failure formed a relatively complete failure band above the gob. The red, blue, and green joint states increased significantly in this stage, indicating intensified incompatible deformation within the surrounding rock, and the interlayer interfaces gradually developed from local dislocation to continuous shear slip and tensile-crack propagation. Compared with the previous two stages, s = 67.0 m was the key stage at which damage evolution changed from slow accumulation to rapid expansion, reflecting significant weakening of the structural integrity of the lower part of the main roof during periodic breakage.

When the advance distance reached s = 80.3 m, the joint-state activation degree within the ROI further increased, and combined slip–tensile failure showed a large-scale continuous distribution above the gob, indicating that the overlying strata had shifted from local rupture to a blocky and discontinuous load-bearing state. This stage corresponded to the stage in the physical similarity simulation when the second sub-key stratum began to show obvious subsidence and forward propagation. As periodic breakage continued to develop, the damage zone beneath the key stratum gradually expanded upward and laterally, and the overburden load-bearing path was further reconstructed.

Overall, the four advance stages show that UDEC joint-state evolution corresponded well with the initial and periodic weighting stages identified by the physical similarity simulation. Overburden breakage was not completed suddenly; instead, it experienced a progressive process of “local joint activation-slip-failure expansion-enhanced tensile failure-combined slip–tensile instability”. The ROI was the most concentrated spatial location of this process. It was not only the technical window for UDEC image statistics, but also the physical window linking structural damage evolution beneath the key stratum with mine-pressure response.

**Figure 11:**
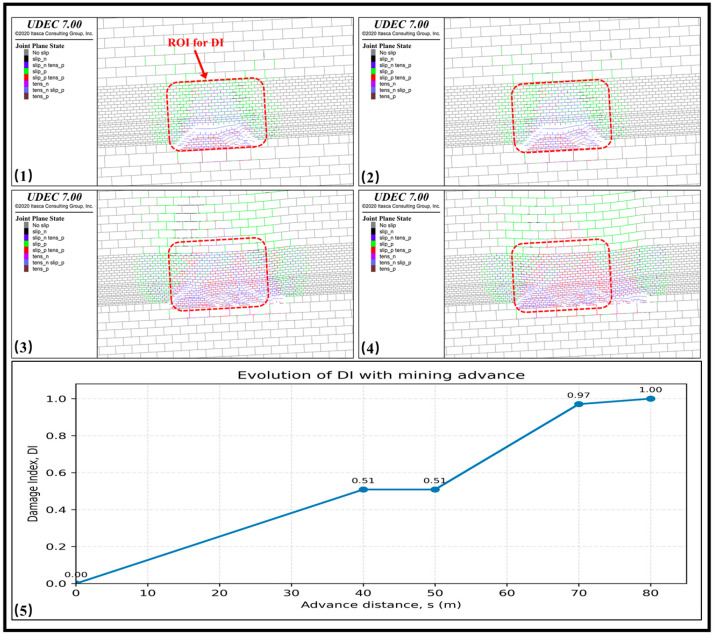
Evolution characteristics of joint states within the ROI at different advance distances: (**1**) s = 37.5 m; (**2**) s = 51.3 m; (**3**) s = 67.0 m; (**4**) s = 80.3 m; and (**5**) evolution of the joint-state-based DI with advance distance. The red dashed box denotes the fixed ROI used for DI calculation, and the colors correspond to the UDEC Joint Plane State legend.

Figure 11 shows that the joint-state-based DI increased in a staged manner with the advance distance. At s = 37.5 m, the DI was approximately 0.51, indicating that obvious joint activation had already occurred within the ROI near the initial weighting stage. At s = 51.3 m, the DI remained close to 0.51, suggesting that the first periodic breakage stage was dominated by the extension and reorganization of the pre-existing damage zone rather than by a new damage jump. When s increased to 67.0 m, the DI rapidly increased to approximately 0.97, showing that slip, tensile opening, and combined slip–tensile failure became concentrated in the ROI. At s = 80.3 m, the DI reached approximately 1.00, indicating the highest damage level in the analyzed sequence. This staged DI growth is consistent with the physical-simulation sequence from initial weighting to periodic breakage and demonstrates that the ROI is the key spatial window connecting overburden damage evolution with mine-pressure response.

**Figure 12:**
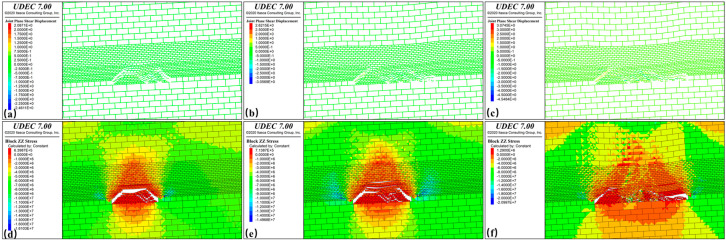
Evolution of shear and stress: (**a**) joint shear-displacement field at s = 37.5 m; (**b**) joint shear-displacement field at s = 67.0 m; (**c**) joint shear-displacement field at s = 80.3 m; (**d**) stress field at s = 37.5 m; (**e**) stress field at s = 67.0 m; and (**f**) stress field at s = 80.3 m. The scientific notation in the legends follows the default UDEC output format, in which “E + n” denotes “×10^n^”; for example, 8E3 denotes 8 × 10^3^.

Figure 12 further provides mechanical support for the DI interpretation. The shear-displacement and stress-response maps show that the stages with rapid DI growth also exhibited enhanced shear slip and stress redistribution around the gob. Therefore, the DI result can be interpreted as a quantitative expression of joint-state transition beneath the key stratum, while the shear-displacement and stress-response results independently support the same damage-evolution process. This combined evidence strengthens the explanation that mine-pressure events are controlled by progressive structural reorganization rather than by random pressure fluctuation alone.

### 4.3. Auxiliary LSTM-Based Identification and Benchmarking of Mine-Pressure Response

The preceding results show that mine-pressure response is not merely a pressure fluctuation, but is mechanically related to key-stratum breakage, joint-state activation, and load-path reorganization. Therefore, the purpose of introducing LSTM in this study is not to establish a universal optimal forecasting model, but to examine whether the support-pressure sequence contains learnable short-term temporal patterns associated with surrounding-rock structural evolution. The data preprocessing, LSTM modeling, and baseline evaluation were implemented in Python 3.12.12 (Python Software Foundation, Wilmington, DE, USA).

On this basis, the historical support-pressure series of the 002Z measuring point was transformed into supervised samples using sliding windows of 24, 36, and 48. A one-step-ahead prediction strategy was adopted, in which the historical pressure sequence within the current window was used to predict the pressure value at the next time step. All models were evaluated using a chronological train–test splitting strategy to avoid future-information leakage caused by random shuffling. The model configuration and chronological data-splitting strategy are summarized in Table 11.

**Figure 13:**
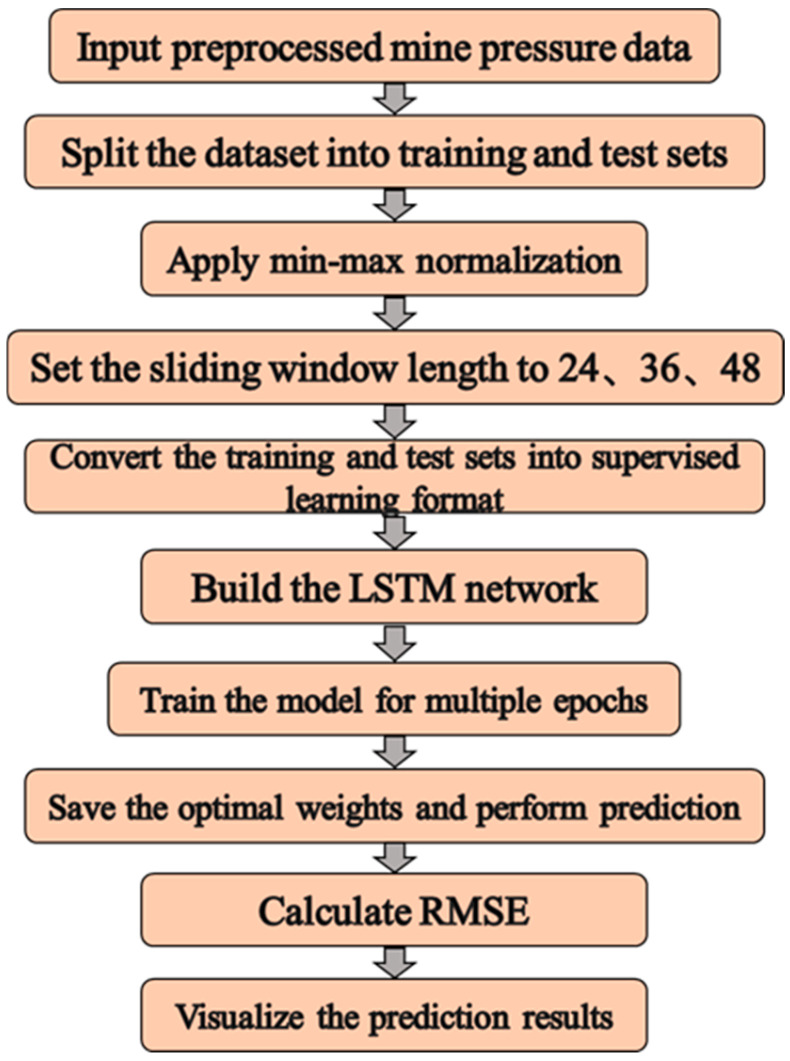
Algorithm flowchart of the sliding-window LSTM mine-pressure prediction model.

The complete workflow of the sliding-window LSTM prediction model is illustrated in Figure 13.

### 4.4. Window-Length Comparison and Baseline Evaluation of Pressure Prediction

Before constructing the LSTM-based mine-pressure prediction model, the 002Z support-pressure data were used for pressure-sequence analysis. Because the field monitoring data contained local missing values and irregular time intervals, interpolation and resampling were performed before model training to obtain a continuous equal-interval support-pressure sequence. To avoid information leakage, the sequence was divided according to chronological order rather than random shuffling. The first 70% of the samples were used for training, and the remaining 30% were used for testing. The prediction horizon was one step ahead. Based on the training configuration used in the LSTM experiments, the number of training epochs was set to 100, the learning rate was set to 0.001, and MSE, RMSE, MAE, and R^2^ were used to evaluate model performance.

**Figure 14:**
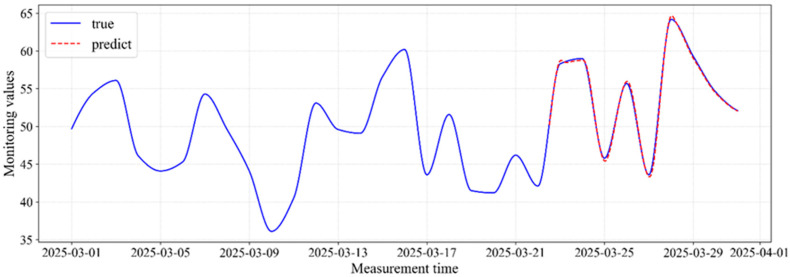
LSTM model results with a sliding-window length of 24.

When the window length was 24, the model had an MSE of 0.0987, RMSE of 0.3141, MAE of 0.2222, and R^2^ of 0.9965. As shown in Figure 14, the blue line represents the processed original pressure data, and the red line represents the model prediction.

**Figure 15:**
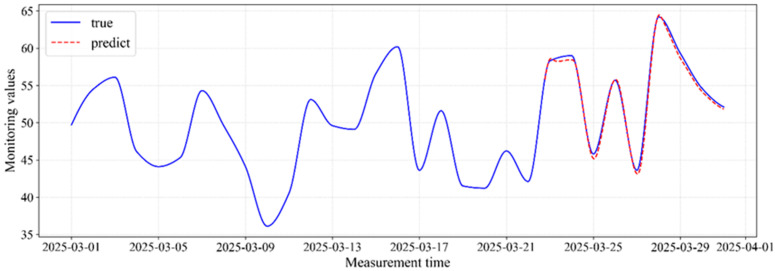
LSTM model results with a sliding-window length of 36.

When the window length was 36, the model had an MSE of 0.2297, RMSE of 0.4792, MAE of 0.4080, and R^2^ of 0.9919. As shown in Figure 15, the blue line represents the processed original pressure data, and the red line represents the model prediction.

**Figure 16:**
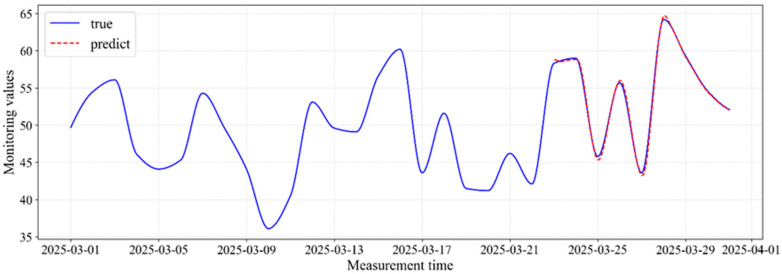
LSTM model results with a sliding-window length of 48.

When the window length was 48, the model had an MSE of 0.1283, RMSE of 0.3583, MAE of 0.2982, and R^2^ of 0.9956. As shown in Figure 16, the blue line represents the processed original pressure data, and the red line represents the model prediction.

The prediction curves further indicate that, when the window length was 24, the model showed good tracking ability for both the overall variation trend and local fluctuations in the test segment, and the deviation between the predicted and actual mine-pressure curves was relatively small. When the window length increased to 36, the model still reflected the overall trend of mine-pressure variation; however, the predicted curve showed some lag and smoothing in locally fluctuating intervals. Its response ability to intervals of rapid DI increase and active mine-pressure events decreased, suggesting that an excessively long historical input may weaken the model’s ability to capture short-term dynamic variation features. When the window length was 48, the model output still reflected the overall variation direction, but its response to local fluctuations was clearly smoother, and its representation of short-term changes and local anomalies weakened. This indicates that, under the current data sample and sequence-structure conditions, a longer window did not provide additional effective information; instead, it may have weakened the model’s response to recent mine-pressure variations.

As shown in Table 12, the experimental results for sliding-window lengths of 24, 36, and 48 were compared. The model achieved the best prediction performance when the sliding-window length was 24, with MSE, RMSE, MAE, and R^2^ values of 0.0987, 0.3141, 0.2222, and 0.9965, respectively. The table indicates that model performance varied considerably under different sliding-window lengths; therefore, selecting reasonable parameters can improve model performance to a certain extent during model training.

Three baseline models were further evaluated using the same 002Z support-pressure sequence and the same chronological train–test split. The persistence baseline was defined as $\hat{P}$_t+1_ = P_t_, the moving-average baseline used the mean value of the previous window, and the random forest model used the same lag-window input as the LSTM model. The benchmark results are summarized in Table 13.

The benchmark comparison shows that the persistence baseline achieved the lowest numerical error because the 002Z pressure sequence was highly continuous and strongly autocorrelated in the short term. Therefore, this study does not claim that the LSTM is an absolutely superior forecasting model under the one-step-ahead prediction setting. Instead, the LSTM is interpreted as an auxiliary temporal-pattern identification model. The 24-step-window LSTM still shows two important features: it produced the lowest error among the tested LSTM window lengths and clearly outperformed the moving-average and random forest baselines, indicating that the pressure series contains learnable short-term patterns beyond simple smoothing or tree-based lag mapping.

### 4.5. Mechanical Consistency Between DI Evolution and Prediction-Error Response

A formal statistical correlation between the DI rate of change and the LSTM prediction error requires the original pointwise prediction-error sequence at the same resolution as the DI evolution sequence. In this study, only the reported LSTM performance metrics and prediction figures were available; therefore, reconstructing a Pearson or Spearman correlation coefficient would be unreliable. Accordingly, the relationship between DI evolution and prediction-error response was interpreted as a mechanical consistency analysis rather than as a statistically verified correlation.

This comparison remains meaningful from a strata-control perspective. When the DI remained low or changed slowly, the ROI was dominated by local slip and deformation accumulation, and the support-pressure sequence was relatively smooth. Under these conditions, the LSTM tracking error remained relatively small. When the DI increased rapidly from approximately 0.51 to 0.97–1.00 between 51.3 and 80.3 m, the ROI entered a stage of concentrated joint-state activation, tensile opening, and combined slip–tensile failure. These structural transitions are mechanically consistent with stronger non-stationarity of the pressure sequence, including local peaks and abrupt fluctuations. Therefore, the increase in prediction deviation near active mine-pressure stages is interpreted as a qualitative consistency between structural damage evolution and pressure-response instability, rather than as independent statistical proof of a DI–error correlation.

In this interpretation, the DI result explains why pressure prediction becomes more difficult during structural activation, whereas the LSTM result indicates that the support-pressure sequence contains learnable short-term temporal patterns. Future work should preserve the pointwise prediction series and calculate Pearson or Spearman coefficients between ΔDI(s) and ∣P_true_ − P_pred_∣ after resampling both variables onto the same advance-distance axis.

## 5. Conclusions

(1) The results of physical similarity simulation and underground monitoring show that overburden-structure evolution during working-face retreat had obvious stages, sequentially manifested as top-coal caving, immediate-roof bed separation and caving, initial main-roof breakage, and periodic breakage. In the physical similarity model, the initial weighting interval was approximately 37.5 cm, and the periodic weighting intervals were mainly distributed within 13.3–15.7 cm, with an average of approximately 14.3 cm. The peak advance abutment pressure was located approximately 9–17 cm ahead of the working face, with a peak coefficient of approximately 1.40–1.68 and an influence range of approximately 36–90 cm. Underground multi-point monitoring at P1-P3 showed good agreement with the physical simulation in terms of peak migration, phase difference, and stage division, indicating that key-stratum breakage and load redistribution are the main controlling factors of mine-pressure manifestation.
(2) The key-stratum deformation-monitoring results show that the deformation growth rate and fluctuation amplitude increased synchronously before initial weighting, and an abrupt jump occurred when initial weighting took place. During periodic weighting, the response was characterized by cumulative subsidence formed by multiple superposed steps. This indicates that key-stratum displacement provides a useful precursor indicator for the transition of surrounding-rock structure from progressive deformation to localized instability and can jointly characterize the occurrence stage of working-face mine-pressure events together with abutment-pressure peaks.
(3) Based on UDEC numerical simulation, joint-state transition, shear slip, and damage activation within a fixed ROI beneath the key stratum were quantified using a joint-state-based damage index (DI). The results show that, as the advance distance increased from 37.5 m to 80.3 m, joints within the ROI developed from local activation to continuous slip, tensile opening, and combined slip–tensile failure. The DI was approximately 0.51 at 37.5 m and 51.3 m, increased rapidly to approximately 0.97 at 67.0 m, and reached approximately 1.00 at 80.3 m. Sensitivity analysis under equal weighting and alternative state-severity weighting schemes showed that the main damage-activation interval and peak stage remained unchanged, indicating that the DI trend was not controlled by an arbitrary weight setting. Combined with the physical-similarity and monitoring results, the rapid DI-growth interval corresponded to periodic weighting and active mine-pressure response, indicating that the ROI beneath the key stratum is the key region linking overburden damage evolution with mine-pressure manifestation.
(4) The LSTM-based pressure-sequence analysis shows that the mine-pressure response contains learnable short-term temporal information associated with surrounding-rock structural evolution. Under the same preprocessing and training settings, the model with a window length of 24 achieved the best performance among the tested LSTM window lengths, with MSE, RMSE, MAE, and R^2^ values of 0.0987, 0.3141, 0.2222, and 0.9965, respectively. Additional benchmark tests showed that the persistence baseline achieved RMSE = 0.2576, MAE = 0.1959, and R^2^ = 0.9979 because of the strong short-term autocorrelation of the pressure sequence, whereas the moving-average and random forest baselines produced higher errors than the 24-step-window LSTM. Therefore, the LSTM is not presented as an exclusive optimal predictor; instead, it is used as an auxiliary temporal-pattern identification module. Combined with the DI results, the increase in prediction deviation near rapid DI-growth stages is mechanically consistent with the non-stationary pressure response induced by joint-state activation and key-stratum structural adjustment. The proposed “multi-source sensing–damage characterization–mechanism verification–sequence identification” framework realizes comparative analysis of physical similarity simulation, field monitoring, numerical simulation, and data-driven results under a unified advance-distance coordinate, providing a reference for mine-pressure anomaly identification and surrounding-rock control in fully mechanized longwall faces.

## Figures and Tables

**Table 1:** Temperature-compensation calibration evidence for the FBG pressure sensor.

Grating	Condition	Fitting Equation	R^2^	Residual Sensitivity Difference After Compensation
FBG1	Constant temperature	y = −0.0352x + 1529.6	0.9992	-
FBG1	Variable temperature before compensation	y = −0.0318x + 1529.6	0.9962	-
FBG1	Variable temperature after compensation	y = −0.0351x + 1529.6	0.9997	0.28%
FBG2	Constant temperature	y = −0.0348x + 1534.9	0.9993	-
FBG2	Variable temperature before compensation	y = −0.0305x + 1534.9	0.9994	-
FBG2	Variable temperature after compensation	y = −0.0346x + 1534.9	0.9991	0.57%

**Table 2:** Pixel classification and weighting rules for the joint-state-based DI.

UDEC State Type	Pixel-Identification Rule	Mechanical Meaning	Weight	Reason for Treatment
No-slip/model grid	Black or gray	Not used in DI	0	Avoids over-counting block boundaries
slip_p	Green-dominant pixels	Persistent shear slip	0.45	Indicates interlayer shear activation
tensile-slip related states	Blue-dominant pixels	Tensile-slip coupled response	0.70	Represents stronger structural opening than pure slip
slip_p + tens_p	Red-dominant pixels	Combined shear–tensile failure	1.00	Highest damage severity
tensile states	Purple/brown-dominant pixels	Tensile opening/tensile activation	0.85	Strong loss of structural continuity

**Table 3:** DI values and physical-model events.

Advance Distance s/(m)	Corresponding Physical-Model Event	DI	Interpretation
37.5	Initial weighting/initial main-roof breakage	0.509	Damage activated but still localized
51.3	First periodic weighting stage	0.509	Existing damage zone extends and reorganizes
67.0	Periodic weighting development	0.970	Rapid transition to concentrated activation
80.3	Strong periodic weighting/sub-key stratum response	1.000	Maximum damage among the selected stages

**Table 4:** Sensitivity analysis of DI under alternative weighting schemes.

Weighting Scheme	DI at 37.5 m	DI at 51.3 m	DI at 67.0 m	DI at 80.3 m	Peak Stage
Default state-severity weights	0.509	0.509	0.970	1.000	80.3 m
Equal active-state weights	0.588	0.588	0.991	1.000	80.3 m
Slip-dominant perturbation	0.544	0.544	0.985	1.000	80.3 m
Tensile-dominant perturbation	0.481	0.481	0.968	1.000	80.3 m

**Table 5:** Similarity rules used in the physical model.

Similarity Item	Expression	Value or Treatment	Role in This Study
Geometry/advance distance	C_l_ = l_p_/l_m_	100	Used to convert model advance distance from cm to prototype m.
Displacement	C_u_ = u_p_/u_m_	100	Used for key-stratum movement scaling when displacement is interpreted.
Unit weight	C_γ_ = γ_p_/γ_m_	Layer/material dependent	Controlled by the prepared similar-material density and prototype lithology.
Stress/load	C_σ_ = C_γ_C_l_	Implemented through material matching and upper loading	Used to satisfy load similarity in the physical model.
Time	Quasi-static advance process	Not used for dynamic time scaling	Weighting intervals were compared by advance distance s, not by elapsed time.

**Table 6:** Explanation of material-ratio codes and components used in the physical model.

Item	Role	Explanation
455, 573, 673, etc.	Similar-material mix code	Relative proportion level of aggregate and cementing materials for a target lithology
Sand	Aggregate	Controls bulk density and granular skeleton of the similar material
Industrial gypsum	Cementing material	Controls early strength and stiffness
Industrial calcium carbonate	Cementing/filling material	Adjusts strength, density, and brittleness
Water	Mixing medium	Ensures material workability during layer construction
Mica powder	Bedding simulation material	Weakens interlayer bonding to simulate stratification

**Table 7:** Material ratio of the physical similarity model.

No.	Mix Code	Lithology	Thickness (cm)	Mass (kg)
1	455	Limestone	1.80	15.30
2	573	Black mudstone	11.30	96.05
3	455	Fine sandstone	6.35	53.98
4	673	Interbedded coal and rock	2.15	18.28
5	537	Limestone	4.95	42.08
6	555	Siltstone	4.95	42.08
7	655	No. 12 coal seam	1.29	10.97
8	373	Fine sandstone	20.93	177.91
9	473	Limestone	2.30	19.55
10	537	Siltstone	12.24	104.04
11	673	Interbedded coal and rock	10.46	88.91
12	337	Siltstone	12.30	104.55
Total	-	-	114.30	970.11

**Table 8:** Caving characteristics of the immediate roof.

Caving Event	Advance Distance /cm	Caving Length /cm	Caving Angle /°
First caving	19	17.5	61
Second caving	26.5	11.2	60
Third caving	33.5	13.5	63
Fourth caving	40.5	7	64

**Table 9:** UDEC model settings used for reproducibility.

Model Item	Setting Used in This Study	Purpose
Software	UDEC 7.00	Discrete-element simulation platform used for block/joint response
Constitutive framework	Block deformation with Coulomb-slip joint behavior	Suitable for layered rock mass with bedding and joint-state evolution
Layer grouping	Lithology-based groups corresponding to the physical model	Fine sandstone, limestone, siltstone, coal seam, mudstone and interbedded coal-rock layers
Excavation scheme	Equal-step excavation; 10 m per step	Used to reproduce progressive longwall advance
Advance reference	Open-off cut as s = 0	Physical model and UDEC aligned by advance distance s
Convergence criterion	Mechanical equilibrium was reached at each excavation stage; the unbalanced force ratio was controlled at approximately 1 × 10^−5^	Consistent with the UDEC solving record
Output variables used	Joint Plane State; Joint Plane Shear Displacement; stress/structural response maps	Joint-state images were used for DI; shear/stress maps were used as mechanical corroboration
ROI treatment	Fixed rectangular ROI above the gob and below the key stratum	Ensures comparable statistics across stages

**Table 10:** Cross-scale correspondence between physical simulation and UDEC stages.

Event	Physical-Model Position	Prototype-Scale Position	UDEC Comparison Stage	Validation Meaning
Initial weighting	37.5 cm	37.5 m	s = 37.5 m stage/nearest UDEC state	Initial main-roof breakage and local joint activation
First periodic breakage	51.3 cm	51.3 m	s = 51.3 m stage/nearest UDEC state	Damage extension and first periodic adjustment
Periodic breakage development	67.0 cm	67.0 m	s = 67.0 m stage/nearest UDEC state	Rapid joint-state activation and large DI increase
Strong periodic response	80.3 cm	80.3 m	s = 80.3 m stage/nearest UDEC state	Continuous slip–tensile failure and maximum DI in the selected sequence

**Table 11:** LSTM model configuration and data-splitting strategy.

Item	Configuration Used in This Study	Purpose
Input sequence	Historical support-pressure series of the 002Z measuring point, [P_t−L+1_, …, P_t_]	Defines the input variable for LSTM and baseline comparison
Sliding-window lengths	24, 36, and 48	Tests the influence of historical-window length
Prediction horizon	One-step-ahead prediction, $\hat{P}$_t+1_, using [P_t−L+1_, …, P_t_]	Clarifies the forecasting horizon
Train/test split	Chronological 70% training and 30% testing; no random shuffling	Avoids future-information leakage from temporal interpolation and random splitting
Validation treatment	Model selection was based on the fixed chronological test segment under identical preprocessing and training settings	Keeps comparison among window lengths consistent
LSTM structure	Two LSTM layers followed by a fully connected output head	Defines model architecture
Hidden units	64	Defines model capacity
Dropout	0.20 between LSTM layers	Controls overfitting in the recurrent layers
Batch size	32	Defines mini-batch training setting
Epochs	100	Consistent with the manuscript training setting
Optimizer/learning rate	Adam optimizer; learning rate = 0.001	Defines optimization setting
Evaluation metrics	MSE, RMSE, MAE, and R^2^	Allows comparison with LSTM and baseline results

**Table 12:** Prediction performance of LSTM under different sliding-window lengths.

Sliding Window Size	24	36	48
MSE	0.0987	0.2297	0.1283
RMSE	0.3141	0.4792	0.3583
MAE	0.2222	0.4080	0.2982
R^2^	0.9965	0.9919	0.9956

**Table 13:** Performance comparison between LSTM models and baseline models for the 002Z support-pressure sequence.

Model	Window/Lag Setting	MSE	RMSE	MAE	R^2^	Interpretation
LSTM	24	0.0987	0.3141	0.2222	0.9965	Best LSTM window
LSTM	36	0.2297	0.4792	0.4080	0.9919	Lower local sensitivity
LSTM	48	0.1283	0.3583	0.2982	0.9956	Weaker than window 24
Persistence baseline	one-step lag	0.0663	0.2576	0.1959	0.9979	Strong autocorrelation
Moving-average baseline	24	9.1529	3.0254	2.3374	0.7104	Over-smoothed
Random forest baseline	24	1.0218	1.0108	0.4628	0.9677	Weaker than LSTM-24

## Data Availability

All data and code used or analyzed in this study are available from the corresponding author on reasonable request.
